# Evaluation of a CT-like Multi-Echo 3D Gradient-Echo MR Sequence (FRACTURE) for Structural Assessment of the Sacroiliac Joints in Spondyloarthritis: Comparison with CT and T1-Weighted MR Imaging

**DOI:** 10.3390/life16101603

**Published:** 2026-09-24

**Authors:** Etienne Leroux, David Fadli, Paolo Spinnato, Nicolas Poursac, Olivier Hauger, Amandine Crombé

**Affiliations:** 1 Department of Musculoskeletal Imaging, Pellegrin University Hospital CHU Bordeaux, 33000 Bordeaux, France; 2 Diagnostic and Interventional Radiology, IRCCS, Istituto Ortopedico Rizzoli, 40136 Bologna, Italy; paolo.spinnato@ior.it; 3 Department of Rheumatology, Pellegrin University Hospital CHU Bordeaux, 33000 Bordeaux, France; 4 Centre de Résonance Magnétique des Systèmes Biologiques (CRMSB), UMR 5536, CNRS, Université de Bordeaux, 146 Rue Léo Saignat, 33076 Bordeaux, France; 5 SARCOTARGET Team, Inserm, UMR1312, BRIC, Bordeaux Institute of Oncology, University of Bordeaux, 33000 Bordeaux, France

**Keywords:** spondyloarthropathy, sacroiliitis, structural lesions, computed tomography, CT-like MRI, multi-echo gradient-echo

## Abstract

CT remains the imaging reference standard for evaluating structural sacroiliac joint (SIJ) lesions in spondyloarthropathy (SpA), but its use is limited by ionizing radiation. This retrospective single-center study evaluated a CT-like multi-echo 3D gradient-echo MRI sequence (FRACTURE) for structural SIJ assessment by comparing it with CT and conventional T1-weighted MR imaging (T1-WI). Consecutive adults who underwent SIJ MRI between November 2023 and October 2024 were included. Four radiologists assessed inflammatory sclerosis, erosions, and joint space changes using a structural severity score (SSS) derived from the SIMACT study and assigned a three-point Likert score for SpA diagnosis. Forty-three patients (median age, 49 years), including 22 with SpA, were analyzed. The area under the ROC curve for diagnostic Likert scores was 0.842 for T1-WI, 0.792 for CT, and 0.767 for FRACTURE (all pairwise comparisons, *p* > 0.05). Considering only definite diagnoses (Likert = 2), diagnostic accuracy reached 81.4% for CT, 76.7% for T1-WI, and 69.8% for FRACTURE, with 100% specificity for all three techniques. FRACTURE detected additional lumbosacral neojoints and accessory articular facets compared with T1-WI and showed a stronger correlation with CT for structural severity assessment (ρ = 0.814 vs. ρ = 0.774). Intra- and inter-observer reproducibility were good (all ICCs > 0.700). In conclusion, FRACTURE provides CT-like structural information with good reproducibility and showed a numerically higher correlation with CT for overall structural severity than T1-WI, while also improving the depiction of certain osseous anatomical variants relevant to differential diagnosis. However, it did not improve diagnostic performance for SpA diagnosis and showed lower performance for erosion assessment. FRACTURE may therefore provide complementary added value within MRI-based SIJ assessment, although further prospective validation is warranted.

## 1. Introduction

Spondyloarthropathies (SpA) are chronic rheumatic diseases primarily affecting the axial skeleton, with an estimated prevalence of 1% in the general population. Diagnosis is based on clinical, biological, and radiological criteria, as outlined by the ASAS guidelines. Early detection is critical for timely treatment initiation to prevent inflammation-driven structural damage progression [1,2,3].

MRI is the preferred imaging modality for diagnosing SpA, assessing disease severity, and monitoring progression, particularly in the sacroiliac joints (SIJs). MRI detects active inflammation, primarily bone marrow edema (BME), using fat-suppressed sequences. It also identifies chronic structural lesions characteristic of SpA, including erosions, subchondral sclerosis, joint space changes, and ankylosis, best visualized on T1-weighted imaging (T1-WI) without contrast or fat suppression. Importantly, subchondral sclerosis may be inflammatory or degenerative, with inflammatory sclerosis typically being diffuse and ill-defined, whereas degenerative sclerosis is more often focal and well-defined and associated with mechanical changes such as osteophytes, geodes, or vacuum phenomenon. Erosions can also be assessed using gradient-echo-based Dixon or volumetric interpolated breath-hold sequences, although their diagnostic performance remains variable depending on the protocol and reader experience. Additionally, MRI is valuable in distinguishing SpA from other causes of SIJ changes, including mechanical/degenerative (accounting for up to 41–44% of cases initially suspected as SpA) and infectious etiologies (0.6–1.3%) [4,5,6,7]. However, overlapping imaging features may lead to diagnostic uncertainty. Furthermore, BME is not SpA-specific and can be observed in asymptomatic individuals, emphasizing the need to integrate structural findings into the diagnostic process [3,8].

CT remains the gold standard for evaluating structural damage. In SpA patients, CT abnormalities are present in 92% of cases [9]. Regarding MRI-based structural evaluation, the SIMACT study demonstrated that T1-WI was more sensitive than radiography for detecting SIJ erosions and joint space abnormalities, reinforcing its role in this assessment [10].

However, CT utilization is constrained by radiation exposure-particularly relevant in young adults-and by the increased logistical complexity and financial burden of dual MRI and CT examinations within an increasingly cost-restricted healthcare system. This has prompted interest in MRI techniques capable of improving structural assessment as a complementary, non-ionizing alternative within a single examination. Recent MRI advancements, including ultra-short and zero echo time (UTE and ZTE, respectively) sequences and reduced echo-train length T1-WI techniques, have improved the visualization of cortical and trabecular bone, offering CT-like detail by leveraging the short T2 signal of bone [11,12]. Other approaches, including susceptibility-weighted imaging (SWI) and MRI-based synthetic CT generated using deep learning, have also shown promising results for the depiction of structural SIJ lesions. SWI has demonstrated improved detection of erosions and sclerosis compared with conventional T1-WI, while deep learning-based synthetic CT has shown higher diagnostic performance than T1-WI for detecting erosions, sclerosis, and ankylosis, with CT as the reference standard [13,14]. These techniques have shown performance comparable to CT and superior to conventional T1-WI for detecting structural lesions in various joints. Notably, they achieve a sensitivity exceeding 90% for detecting SIJ erosions, joint space alterations and sclerosis [15,16], with overall sensitivity and specificity for structural lesions reported at 76% and 98.6%, respectively [17].

However, previous studies have primarily focused on sensitivity and specificity for individual structural lesion detection, with limited data on reproducibility and severity scoring. The reproducibility and diagnostic value of CT-like sequences in SpA, as well as their ability to assess structural severity, remain underexplored. Furthermore, there is a lack of distinction between degenerative and inflammatory SIJ sclerosis [7,9,10].

The FRACTURE sequence (‘fast field echo resembling a CT using restricted echo spacing’, developed by Philips Healthcare) is a high-resolution 3D gradient-echo (GRE) technique designed to enhance bone delineation and minimize signal loss at tissue interfaces. It uses multiple echoes and post-processing, including echo summation and grayscale inversion, to generate CT-like bone contrast while preserving the advantages of MRI [18]. Its potential has been demonstrated in spine, skull, and shoulder imaging, but its role in SpA remains unstudied [18,19,20].

Thus, this study aimed to evaluate the diagnostic performance of FRACTURE compared to conventional T1-WI and CT for diagnosing SpA-related sacroiliitis based on structural alterations. Rather than assessing its ability to replace CT, we explored its potential role as a complementary imaging tool within MRI protocols. We hypothesized that FRACTURE may provide comparable structural information to CT and complementary information to T1-WI. Secondary objectives included assessing its utility in grading lesion severity using a scoring system derived from the SIMACT study [10], with CT as the reference standard, and evaluating its reproducibility.

## 2. Materials and Methods

### 2.1. Study Design

This single-center, observational retrospective study was approved by the institutional review board of Bordeaux University Hospital (France, approval number: CER-BDX-2024-340) and conducted in full accordance with applicable laws, regulations, and the Declaration of Helsinki.

Inclusion criteria were: (i) age ≥ 18 years, (ii) SIJ MRI performed at Bordeaux University Hospital between November 2023 and November 2024 for pelvic/lower back pain suspicious of sacroiliitis, including turbo spin echo (TSE) T2-WI (with and without fat suppression regardless of the technique), TSE T1-WI, and FRACTURE sequence, all acquired in the oblique coronal plane of the SIJs, (iii) availability of a CT, including the pelvis in bone kernel, performed within the past 3 months and stored in our Picture Archiving and Communication System (PACS), and (iv) final diagnosis (inflammatory or degenerative) established by the multidisciplinary board of our hospital (comprising musculoskeletal radiologists, rheumatologists, orthopedic surgeons, and an internal medicine specialist).

Exclusion criteria were non-diagnostic quality imaging (CT or MRI) and no clearly established final rheumatologic diagnosis.

The primary objective of this study was to compare the diagnostic performance of FRACTURE, CT, and T1-weighted imaging (T1-WI) for the positive diagnosis of SpA-related sacroiliitis, using the diagnosis made by the multidisciplinary board as the reference standard.

Given the exploratory nature of this study, no formal superiority hypothesis was predefined. However, sample size considerations were informed by prior studies [20], assuming a CT accuracy of 85% and an accuracy of CT-like sequences of approximately 81%. Under these assumptions, a minimum of 21 patients per group would provide 80% power with a one-sided alpha level of 10% and a non-inferiority margin (delta) of 15%, as estimated using the power.prop.test function in R (v4.1.1, The R foundation for statistical computting, Vienna, Austria). These parameters were used to guide feasibility rather than to formally test a predefined non-inferiority hypothesis.

Figure 1 shows the study flow-chart and statistical workflow.

### 2.2. Non-Radiological Data Collection

Clinical data were retrieved from hospital medical records, including age, sex, body mass index (BMI), C-reactive protein (CRP), HLA-B27 status, and the final conclusion of the multidisciplinary board (degenerative SIJ changes or inflammatory sacroiliitis). Inflammatory sacroiliitis was defined according to established ASAS recommendations, primarily based on the presence of active inflammatory lesions, clearly detectable subchondral bone marrow edema/osteitis on MRI (with associated features such as synovitis, capsulitis, or enthesitis considered in the overall assessment) [21]. Structural lesions, including erosions, sclerosis, fat metaplasia, and ankylosis, were considered supportive of spondyloarthritis but were not sufficient on their own to define active sacroiliitis. Degenerative SIJ changes were defined as non-inflammatory structural abnormalities, including joint space narrowing, subchondral dense sclerosis, osteophyte formation, and irregularity or remodeling of the articular margins, without active inflammatory lesions characteristic of sacroiliitis. The reference standard corresponded to this final diagnosis, established through integration of clinical, biological, and imaging findings (including both inflammatory and structural features), as well as follow-up data. Consequently, some patients classified as SpA did not necessarily exhibit structural sacroiliac lesions at the time of imaging. In patients with rheumatologic sacroiliitis, additional data on peripheral arthritis, disease duration, and current treatment were collected.

### 2.3. Image Acquisitions

CT acquisitions were performed on various 64- or 128-detector row CT-scanners from our center and external centers. The slice thickness ranged between 0.60 and 1.25 mm. The in-plane resolution ranged between 0.35 × 0.35 and 0.85 × 0.85 mm^2^.

MRI acquisitions were all performed on the same 1.5-Tesla MR-system (Ingenia Ambition X, Philips Medical Systems, Best, The Netherlands). 

The routine protocol for SIJ imaging comprised coronal-oblique TSE DIXON T2-WI (with Fat, Water and In images) or spectral attenuated inversion recovery (SPAIR) T2-WI, coronal-oblique TSE T1-WI and coronal-oblique FRACTURE.

Regarding the technical implementation of CT-like MRI approaches, while UTE and ZTE techniques enable direct signal acquisition from short T2 tissues and can generate CT-like images, their clinical implementation remains limited by specific hardware and sequence requirements, including rapid transmit–receive switching, high-performance gradients, and dedicated pulse programming, which restrict their availability across MRI systems [11]. Other techniques, such as ‘sweep imaging with Fourier transform’ (SWIFT) or fast large-angle spin echo (FLASE), have also been proposed but similarly require advanced sequence design [11]. In contrast, 3D GRE-based approaches offer a more widely accessible alternative, as they can be implemented on most clinical MRI platforms while providing high spatial resolution and robust bone–soft-tissue contrast [11,16]. These include multi-echo GRE acquisitions and ‘black bone’ imaging, which exploit differences in tissue signal properties and allow post-processing to enhance bone depiction. In this context, the FRACTURE sequence (‘fast field echo resembling CT using restricted echo spacing’) corresponds to a high-resolution 3D multi-echo spoiled GRE acquisition with short repetition time and low flip angle, enabling efficient sampling of short T2 tissues [16]. The sequence acquires multiple echoes (*n* = 6 echoes) at constant echo spacing (ΔTE ≈ 4.6 ms), reducing chemical shift artifacts and signal loss. Post-processing includes echo summation to improve signal-to-noise ratio, followed by subtraction and grayscale inversion, resulting in CT-like bone contrast. Although implemented here using a vendor-specific solution (Philips Healthcare, Best, the Netherlands), this approach belongs to a broader class of GRE-based CT-like MRI techniques available across vendors [11].

All acquisitions were performed using the spine antenna (32 elements) and the body antenna (16 elements).

Table 1 summarizes the characteristics of the sequences.

### 2.4. Image Reading

Four radiologists participated in the study: 3 seniors specialized in musculoskeletal imaging (D.F., A.C. [or Reader-1] and O.H., with 3, 6 and 20 years of experience, respectively) and one radiology resident (E.L. [or Reader-2], with 6 months of residency in our department). 

The assessment for structural damages was derived from the analysis grid proposed by Diekhoff et al. [10] but differed regarding the definition of erosion and the division of the SIJ. Indeed, each SIJ was assessed separately (i.e., right and left) and was divided into 3 anatomical positions (anterior [i.e., anterior to the slices depicting the sacral neuroforamina with <180° of the circumference of S2 visualized and the true pelvis seen in the center of the image], middle [defined by the depiction of the anterior sacral foramina] and posterior [recognized by visualization of the entheseal joint compartment in the middle, and extending to the posterior and inferior aspect of the joint]). For each position, three structural items were scored on each type of imaging (T1-WI, FRACTURE and CT):

- Erosions (defined as ill-defined bone defect with a broad base), categorized as: no erosion (0), 1–2 small isolated erosions or questionable single erosion (1), 3–5 definite erosions < 3 mm or larger single erosion (>3 mm) (2), and >5 or confluent erosions (3);
- Sclerosis related to inflammatory disorder defined as diffuse, ill-defined bone densification blending with adjacent bone, usually associated with erosions. On the contrary, degenerative sclerosis, defined as well-defined, focal, dense, and confined to mechanical stress areas, and usually associated with osteophytes, geodes, and vacuum phenomena, was excluded from the analysis. Inflammatory sclerosis was subsequently categorized as: absent (0), questionable (1), and evident (2);
- Joint space alterations, categorized as: no change (0), questionable joint space change (1), pseudowidening (2), partial ankylosis (3), and extensive/total ankylosis (4).

The scores for erosions, sclerosis and joint space alterations were then summed across the 3 positions, which provided a structural severity score (SSS) per SIJ (right or left) and per image type, which could range between 0 and 27 (=3 × 3 [erosion score] + 4 × 3 [joint space score] + 2 × 3 [sclerosis score]).

In addition, the following radiological features related to degenerative SIJ changes were collected on each side and for each image type: lumbosacral neojoints and accessory articular facet.

The last point of the radiological analysis consisted of a Likert score for sacroiliitis related to spondyloarthropathy, ordered as follows: 0: certainly not, 1: doubtful, and 2: strongly suspicious of spondyloarthropathy sacroiliitis. This Likert score was evaluated for each sequence.

Figure 2 represents the collected radiological features on each image type.

The readings were organized as follows (Figure 1): 

Reader-1 and Reader-2 independently reviewed the FRACTURE and T1-WI images, blinded to the other radiologist’s readings and to clinical diagnosis. Each reader performed two separate reading sessions for each sequence (T1-WI and FRACTURE), allowing assessment of intra-observer reproducibility.

The reading workflow was structured in four time-separated sessions with washout periods to minimize recall bias: 1st reading of T1-WI for Reader-1 and FRACTURE for Reader-2, 2 weeks delay, 1st reading for FRACTURE for Reader-1 and T1-WI for Reader-2, 4 weeks delay, 2nd reading of T1-WI for Reader-1 and FRACTURE for Reader-2, 2 weeks delay, and 2nd reading of FRACTURE for Reader-1 and T1-WI for Reader-2.

Four weeks after these individual readings, a consensus review of the FRACTURE, T1-WI, and CT images was conducted by A.C., E.L., D.F. and O.H. (blinded to the clinical diagnosis). In case of disagreement, O.H. helped in finding a definitive consensus. 

It must be noted that DIXON/SPAIR T2-WI were not available during the reading sessions as our study strictly focused on structural damages.

### 2.5. Statistical Analysis

Statistical analyses were performed with R (v4.1.1, The R foundation for statistical computting, Vienna, Austria) (Figure 1). A *p*-value < 0.05 was considered statistically significant. 

*Diagnostic performance for SpA-related sacroiliitis (whole population)*: Diagnostic performance analyses were conducted in a comparative and exploratory framework. This analysis was based on the consensus reading. The reference was the diagnosis from the multidisciplinary board. The area under the ROC curve (AUROC) for the T1-, FRACTURE- and CT-based Likert scores was estimated with a 95% confidence interval (95%CI), and pairwise comparisons were achieved using Delong tests. The odds ratio (OR), accuracy, sensitivity and specificity with 95%CI were estimated in two settings: (i) considering positive Likert scores of 1 and 2, and (ii) considering positive Likert scores of 2. Comparisons of paired proportions (including accuracy) between imaging modalities were performed using McNemar tests. Non-inferiority was not formally tested but explored descriptively through comparative performance metrics. Patient-level comparisons of the rates of agreement between the consensual Likert scores assessed on (i) CT and T1-WI and (ii) CT and FRACTURE were performed using the McNemar test.

*Severity Assessment of sacroiliitis (SpA patients):* This sub-analysis was based on the consensus reading and focused only on patients with a confirmed diagnosis of SpA. Right and left SIJs were analyzed independently to double the number of observations. The SSS assessed with CT was considered as the reference. After verifying the non-normality of SSS using the Shapiro–Wilk normality test, correlations between SSSCT and SSST1-WI and between SSS_CT_ and SSS_FRACTURE_ were assessed using the Spearman rank correlation test. Correlation coefficients (rho) values (ranging from −1 [opposite perfect correlation] to +1 [perfect correlation], with 0 indicating no correlation) were estimated and compared using bootstrapping on 1000 replicates of the population. 

Regarding the subcomponents of the SSS (erosions, sclerosis and joint space), the exact agreement rates between CT and T1-WI, and between CT and FRACTURE were estimated and compared using McNemar tests.

*Intra- and inter-observer reproducibility analysis (whole population):* The intra-class correlation coefficient (ICC) was used for continuous variables, and the weighted Kappa (Kw) for ordinal variables. Intra-observer reproducibility was calculated separately for each reader based on the two independent reading sessions for each sequence, and results were then averaged across readers. Inter-observer reproducibility was derived from the first reading session only to ensure independence between measurements.

*Differential diagnoses (whole population):* This sub-analysis was based on the consensus reading and included all patients. Right and left SIJs were analyzed independently. 

## 3. Results

### 3.1. Patient Characteristics 

Table 2 summarizes the patient characteristics. Forty-three patients with low back/pelvic pain suspicious of sacroiliitis were finally included (20 women [46.5%]). Median age was 49 years (interquartile range [IQR]: 37–52, range: 19–78). Diagnoses were degenerative SIJ changes for 21/43 (48.8%) patients and inflammatory sacroiliitis for 22/43 (51.2%) patients, with a majority being ankylosing spondylitis (20/22, 90.9%). 

There were no significant differences in terms of age and sex between the two groups (*p* = 0.5758 and *p* = 0.8701, respectively—Table 3).

### 3.2. Performances of FRACTURE for Diagnosing SpA-Related Sacroiliitis in the Whole Population

Likert scores were significantly associated with the final diagnosis for all imaging modalities (FRACTURE: *p* = 0.0013; T1-WI and CT: *p* < 0.0001) (Figure 3A, Appendix A).

Using the three-level Likert scale, AUROCs were 0.767 (95%CI = 0.649–0.886) for FRACTURE, 0.792 (95%CI = 0.671–0.913) for CT and 0.842 (95%CI = 0.736–0.948) for T1-WI (Figure 3B)—with no significant differences between imaging modalities (all *p* > 0.05).

After dichotomizing the Likert scores (Table 4), the highest diagnostic accuracy was achieved with CT and T1-WI (81.4%, 95%CI = 66.6–91.6—using a Likert = 2 as predictive of inflammatory sacroiliitis), while FRACTURE reached a maximal accuracy of 74.4% (95%CI = 58.8–86.5) using a Likert ≥ 1 as predictive of inflammatory sacroiliitis. 

Differences in accuracy between modalities were not statistically significant (all *p* > 0.05, Appendix B). 

The highest sensitivity was observed with T1-WI (72.7%, 95%CI = 49.8–89.3—using a Likert ≥ 1 as predictive of inflammatory sacroiliitis), whereas specificity reached 100% for all modalities when considering Likert = 2 as positive; however, this estimate should be interpreted cautiously given the limited sample size and wide confidence intervals (95%CI: 83.9–100).

At the patient level, the consensual Likert-based radiological conclusions were concordant between CT and T1-WI in 36/43 (83.7%) patients and between CT and FRACTURE in 32/43 (74.4%) patients, with no significant difference between modalities (*p* = 0.2888).

As the SSS were evaluated blindly on all patients, the ROC analysis was replicated for the SSSs as a continuous diagnostic biomarker, which provided in ascending order: AUROC = 0.878 (95%CI = 0.765–0.990) for FRACTURE, 0.865 (95%CI = 0.750–0.980) for T1-WI and 0.834 (95%CI = 708–0.961) for CT, without significant differences (all *p* > 0.05). According to the closest top-left method, optimal cut-offs were: 8/64 for SSS_FRACTURE_ (sensitivity = 0.773, specificity = 0.952), 3/64 for SS_T1-WI_ (sensitivity = 0.864, specificity = 0.857) and 15/64 for SSS_CT_ (sensitivity = 0.727, specificity = 0.952) (Figure 4).

Over the 86 SIJs included in the study, 17/86 (19.6%) and 11/86 (12.8%) joints demonstrated accessory articular facets and lumbosacral neojoints, respectively, on reference CT. Regarding accessory articular facets, the agreement between CT and T1-WI was 69.8% (60/86) vs. 72.1% (62/86) between CT and FRACTURE (*p* = 0.5165). Regarding lumbosacral neojoints, the agreement between CT and T1-WI was 87.2% (75/86) vs. 89.5% (77/86) between CT and FRACTURE (*p* = 0.9432). FRACTURE detected two additional lumbosacral neojoints and two additional accessory articular facets compared to T1-WI. Figure 5 shows examples of the potential causes of sacroiliac pain on the different image types.

### 3.3. FRACTURE Performances for Assessing the Severity of Sacroiliitis in SpA Patients

This analysis included 44 SIJs from the 22 SpA patients (Table 5).

Overall, correlation with CT-based SSS was slightly higher for FRACTURE (rho = 0.814, 95%CI: 0.737–0.954) than for T1-WI (rho = 0.774, 95%CI: 0.668–0.988), although this difference was not statistically significant (*p* = 0.7850) (Appendix C).

At the subcomponent level, FRACTURE showed higher correlations with CT for sclerosis (rho = 0.679, 95%CI = 0.474–0.902 vs. 0.503, 95%CI = 0.323–0.710 for T1-WI) and joint space changes (rho = 0.715, 95%CI = 0.546–0.964 vs. 0.562, 95%CI = 0.350–0.830 for T1-WI). In contrast, correlation for erosions was lower with FRACTURE (rho = 0.526, 95%CI: 0.253–0.769) compared with T1-WI (rho = 0.714, 95%CI: 0.555–0.991).

In more detail, the erosion score on T1-WI exceeded the CT-based reference score in 15/44 (34.1%) sacroiliac joints, compared with 11/44 (25.0%) for FRACTURE, suggesting a slight tendency toward overestimation with T1-WI. Similarly, the proportion of sacroiliac joints with at least one erosion was 30/44 (68.2%) on CT, 34/44 (77.3%) on T1-WI, and 32/44 (72.7%) on FRACTURE. However, agreement with CT did not differ significantly between T1-WI and FRACTURE (McNemar test, *p* = 0.4795).

These findings suggest that, while FRACTURE provides good overall agreement with CT for global severity assessment, its performance may be more limited for the evaluation of specific lesion types, particularly erosions.

Figure 6 illustrates the correspondence between the three image types in SpA patients.

### 3.4. Reproducibility Analysis in the Whole Population (Table 6, Appendix D)

Overall, both T1-WI and FRACTURE demonstrated good inter- and intra-observer reproducibility for SSS and Likert scores.

For SSS, inter-observer agreement was similar between T1-WI (ICC = 0.759, 95%CI: 0.578–0.869) and FRACTURE (ICC = 0.741, 95%CI: 0.551–0.859). Intra-observer agreement was slightly higher for T1-WI (ICC = 0.864, 95%CI: 0.754–0.928) compared with FRACTURE (ICC = 0.831, 95%CI: 0.703–0.908).

For Likert scores, inter-observer agreement was substantial for both modalities (T1-WI: Kw = 0.799, 95%CI: 0.633–0.975; FRACTURE: Kw = 0.762, 95%CI: 0.613–0.960), with similarly high intra-observer agreement (T1-WI: Kw = 0.812, 95%CI: 0.659–0.971; FRACTURE: Kw = 0.766, 95%CI: 0.619–0.933).

Overall, reproducibility was comparable between modalities, with a consistent trend toward slightly higher agreement for T1-WI.

## 4. Discussion

In this study, the FRACTURE sequence was compared to T1-WI and CT for the diagnosis and severity assessment of SpA-related structural lesions. FRACTURE showed promising structural assessment performance and may provide complementary information alongside T1-WI and CT, rather than serving as a standalone substitute.

First, considering a Likert score of 2 as predictive of SpA diagnosis based on structural alterations of the SIJs (which would correspond to clinical practice where only strong suspicions would be treated), the highest accuracy was reached with CT (81.4%), followed by T1-WI (76.7%) and FRACTURE (69.8%). All sequences demonstrated a specificity of 100%. Furthermore, at the patient level, the consensual radiological conclusions showed a higher concordance between CT and T1-WI than between CT and FRACTURE, although the difference was not statistically significant. However, we observed a higher AUROC for the Likert score with T1-WI (0.842) compared to CT (0.792) and FRACTURE (0.767). This finding partly contrasts with previous research, which found that CT outperformed conventional MRI, demonstrating comparable sensitivity (76% vs. 82%) but significantly higher specificity (97% vs. 87%) [10].

The good performance of T1-WI in our study could be attributed to the radiologists’ greater familiarity with this sequence, its ability to depict subchondral fatty bone marrow conversion as a sequela of prior active sacroiliitis, and its high resolution, enhanced by artificial intelligence and compressed sensing techniques. However, official diagnostic criteria do not rely solely on structural lesions but also incorporate inflammatory changes, as defined by the ASAS criteria. In line with this, the reference standard in our study was based on a multidisciplinary board integrating clinical, biological, imaging (including both inflammatory and structural findings), and follow-up data. As a result, some patients classified as SpA may not have exhibited structural sacroiliac lesions at the time of imaging, particularly in early disease stages, which may partly explain the moderate performance of imaging modalities focused on structural assessment.

Interestingly, though not designed for diagnosing SpA but for evaluating disease severity, we observed high diagnostic performance of the SSS, especially when it was based on FRACTURE (AUROC = 0.878) compared to CT (AUROC = 0.834) and T1-WI (AUROC = 0.865).

One advantage of FRACTURE was its ability to detect lumbosacral neojoints and accessory articular facets not visible on T1-WI. These anatomical variants can be sources of low back pain and may mimic SpA clinically and radiologically [4]. 

In addition, FRACTURE demonstrated better correlation with CT than T1-WI for lesion severity assessment. Our study used a modified version of the Diekhoff scoring system, particularly by reducing the number of quadrants analyzed and distinguishing mechanical/degenerative vs. inflammatory sclerosis. While FRACTURE was effective in detecting most structural lesions, it was less sensitive than T1-WI for identifying erosions and struggled to differentiate erosions from degenerative subchondral cysts. This finding contrasts with previous studies where CT-like MRI sequences generally exhibit high sensitivity for erosion detection, often surpassing T1-WI. For example, Deppe et al. reported an 82% sensitivity for erosion detection using a CT-like SWI sequence compared to 73% for T1-WI [21]. Similarly, Hahn et al. found that UTE sequences achieved a sensitivity of approximately 80%, consistently outperforming T1-WI [14]. Likewise, Lin et al. reported a sensitivity of 92% for ZTE vs. 81% for T1-WI [22]. In our study, agreement with CT was higher for T1-WI than for FRACTURE, although this difference was not statistically significant. Notably, T1-WI also identified more erosions than CT, suggesting a tendency toward overestimation, whereas FRACTURE appeared more conservative. However, CT (used as the reference standard) may also underestimate very small or early erosions, which should be considered when interpreting these findings. Several factors may explain this discrepancy. First, despite its higher spatial resolution (0.39 × 0.39 mm; 0.55 mm isotropic voxels), FRACTURE relies on a highly accelerated 3D multi-echo gradient-echo acquisition (SENSE factor 7, TR = 30 ms, low flip angle), which may increase image noise and reduce contrast between cortical bone and adjacent structures, limiting detection of subtle cortical defects. In contrast, T1-WI, based on a spin-echo acquisition, provides higher intrinsic contrast and smoother image appearance, facilitating visual detection, albeit with a risk of overcalling. Second, reader experience likely contributed, as radiologists are more familiar with T1-WI than with CT-like sequences such as FRACTURE. Finally, although FRACTURE enables multiplanar reconstructions, these may appear less sharp than directly acquired oblique coronal T1-WI images, potentially reducing lesion conspicuity. However, our results align with prior research regarding sclerosis and sacroiliac joint space narrowing, where FRACTURE and CT-like sequences have demonstrated superiority over T1-WI.

The FRACTURE sequence appeared slightly less reproducible than T1-WI, likely due to its novelty and the radiologists’ limited experience interpreting it. However, its 3D acquisition allows for reconstruction in any plane, which can be advantageous in cases where the standard oblique coronal positioning is suboptimal or when sacroiliac joint morphology is atypical. 

One major advantage of FRACTURE is that it is a non-ionizing technique, making it relevant for young adults with SpA, who require repeated imaging over time. Rather than replacing CT, FRACTURE may help reduce the need for CT in selected cases by providing additional structural information within the MRI examination, particularly when combined with T1-WI. However, the FRACTURE sequence is highly susceptible to magnetic susceptibility artifacts, which can hinder interpretation in the presence of air (as seen in degenerative SIJs) or metallic implants (hip or lumbosacral hardware). 

Compared with other CT-like MRI techniques, FRACTURE relies on a high-resolution 3D multi-echo spoiled GRE acquisition with post-processing to generate CT-like bone contrast. UTE and ZTE directly capture signal from short T2 tissues, enabling excellent cortical bone depiction and high sensitivity for SIJ structural lesions, often approaching CT performance [13,14,20]. However, their broader clinical use remains limited by specific hardware and sequence requirements, including fast transmit–receive switching and dedicated implementations [11]. In contrast, GRE-based approaches such as FRACTURE can be implemented on more conventional MRI systems and more easily integrated into routine protocols. While UTE/ZTE benefit from direct bone signal acquisition, GRE-based techniques rely on indirect contrast and post-processing, making them more sensitive to noise, susceptibility artifacts, and reduced bone–marrow contrast. This may partly account for the lower agreement with CT for erosions observed in our study compared with prior UTE/ZTE reports. Conversely, our findings for sclerosis and joint space assessment are consistent with previous studies showing good performance of CT-like MRI techniques for macroscopic structural changes [13,15]. Hence, FRACTURE should be considered within the spectrum of CT-like MRI approaches, with a favorable trade-off between accessibility and performance. Its main contribution may lie in its feasibility within routine clinical workflows, rather than in outperforming more technically demanding sequences such as UTE or ZTE.

Overall, SSS could be valuable for establishing a baseline for disease monitoring, tracking progression, and assessing treatment efficacy. Indeed, prior studies have suggested that severe radiographic involvement is a predictor of future disease progression, potentially influencing therapeutic strategies. It is therefore reasonable to expect that CT-like sequences could enhance prognostication with greater accuracy [23,24]. Table 7 summarizes the main advantages and limitations of the FRACTURE sequence identified in the present study.

This study has limitations. It was a retrospective study with a relatively small sample size, which limits the precision and generalizability of the findings, as reflected by the wide confidence intervals of several performance estimates. Although sample size considerations were informed by a non-inferiority framework, no formal non-inferiority hypothesis was prespecified or tested, and the observed differences should therefore be interpreted descriptively. This modest sample size may have limited the statistical power of the paired comparisons, including the McNemar tests used to compare diagnostic accuracy between imaging modalities. Some patients in the SpA group had no structural involvement. Our results regarding SpA diagnosis and severity assessment might have been more robust and accurate if the protocol had included spinal imaging. CT reproducibility was not assessed, which may limit direct comparison of reproducibility across imaging modalities. Additionally, we relied solely on radiological scoring without incorporating clinical severity assessments. The use of a simplified three-position adaptation of the SIMACT scoring system, rather than the original 24-region approach, may limit direct comparability with studies using the original scoring method. Notably, considering the right and left SIJs as separate observations may have introduced within-patient dependence and should therefore be considered when interpreting the exploratory joint-level analyses. Furthermore, the use of bootstrap resampling may introduce additional uncertainty due to dependence on sample composition and should be interpreted as exploratory. In addition, the binarization of Likert scales, although facilitating clinical interpretation, may have led to a loss of granularity and reduced sensitivity to subtle differences between imaging types. Moreover, we did not systematically assess the full spectrum of normal anatomical SIJ variations, as our analysis focused on lumbosacral neoarticulations and accessory articular facets considered potentially relevant to the differential diagnosis of low back or sacroiliac pain. Lastly, the heterogeneity of CT acquisition protocols, particularly the wide range of slice thicknesses (0.60–25 mm), may have affected the reference standard and the correlation analyses. In particular, lower spatial resolution with thicker reconstructions may have limited the detection of small erosions, potentially influencing the observed agreement and correlation of MRI techniques with CT.

## 5. Conclusions

The FRACTURE sequence demonstrated comparable diagnostic performance to CT in this exploratory study and showed good correlation with CT for severity assessment, while remaining complementary to conventional MRI sequences. However, its lower performance for erosion detection and the exploratory nature of this study warrant cautious interpretation. Nevertheless, it is a promising technique that could aid in SpA diagnosis without additional cost. Further prospective studies with larger cohorts are required to better define its role within multimodal imaging strategies, rather than as a replacement for CT.

## Figures and Tables

**Figure 1:**
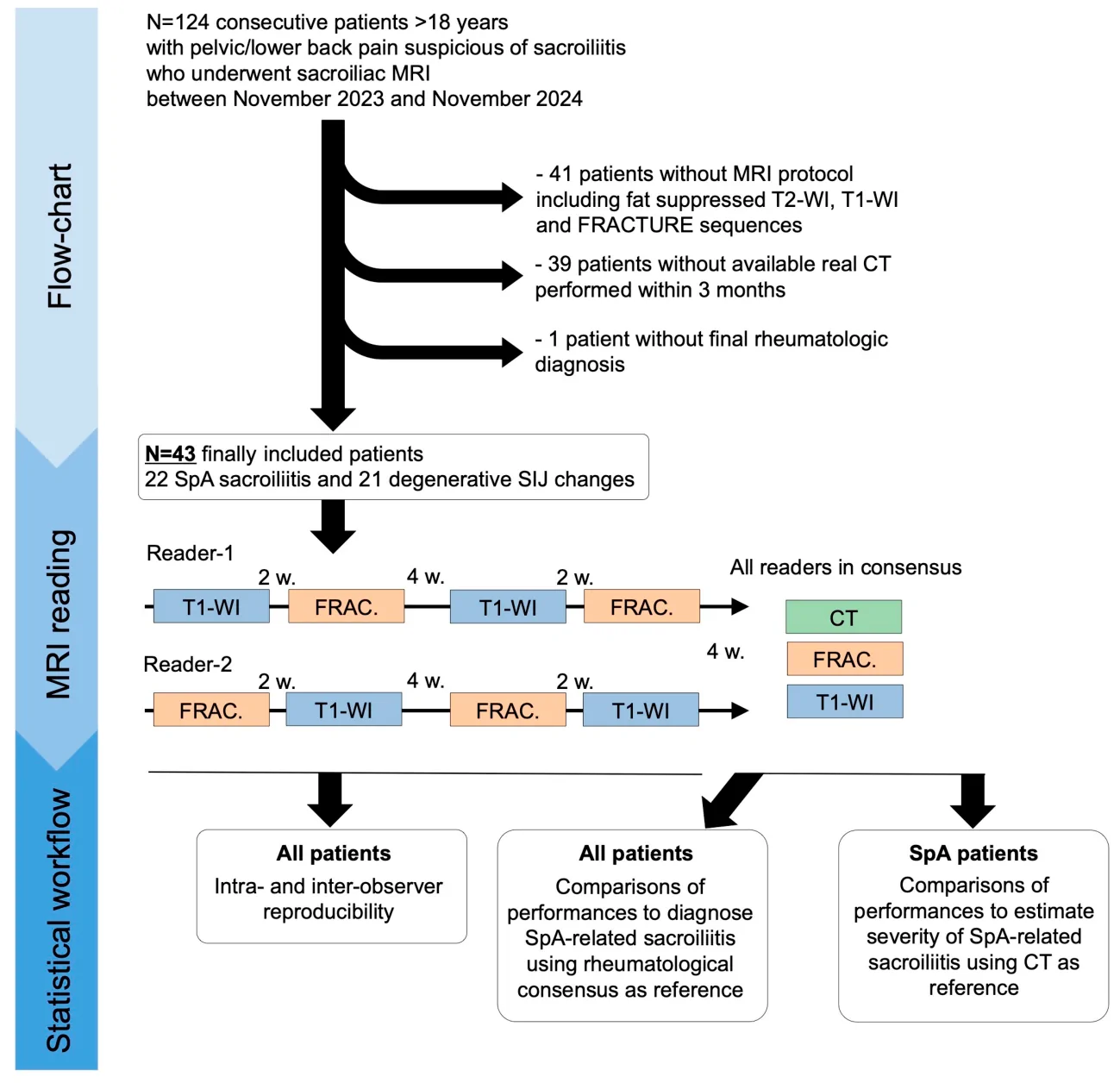
Study flow-chart, imaging reading organization and statistical workflow. The study was designed to include a minimum of 21 patients per group given the primary objective. Abbreviations: CT: computed tomography, FRAC.: FRACTURE sequence, SIJ: sacroiliac joint, SpA: spondyloarthropathy, w.: weeks, WI: weighted imaging.

**Figure 2:**
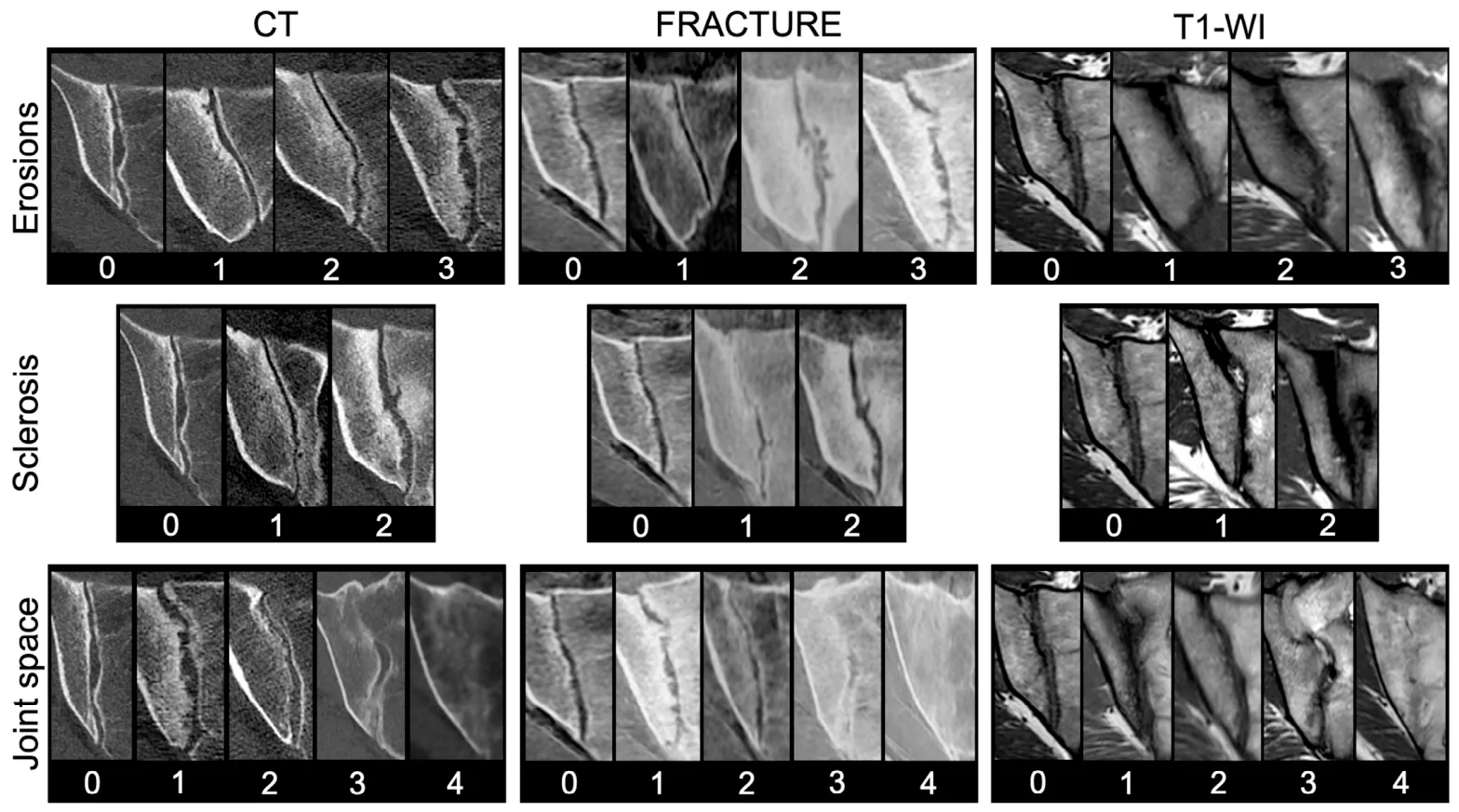
Scoring atlas for the 3 evaluated imaging according to the system proposed by Diekhoff et al. including a score for erosions, sclerosis and joint space (as described in the method) [10]. Abbreviations: WI: weighted imaging.

**Figure 3:**
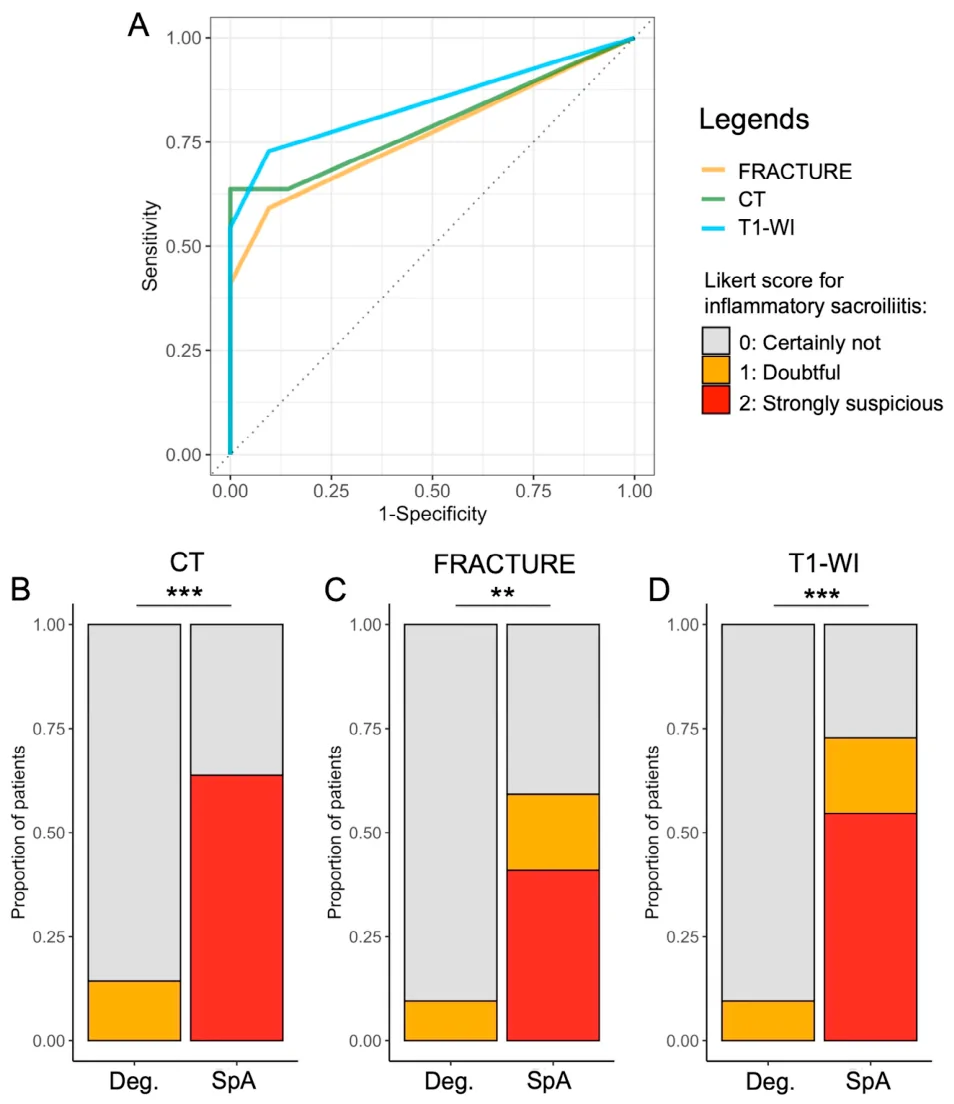
Performance of the consensus Likert score assessed on FRACTURE, CT and T1-weighted imaging (WI) to diagnose inflammatory sacroiliitis. (**A**) ROC curve for the 3 Likert scores. Barcharts showing the distribution of the 3 categories of the Likert score depending on the patient groups (i.e., spondyloarthropathy [SpA] and mechanical/degenerative [Deg.] sacroiliac joint changes) and the image type, i.e., CT (**B**), FRACTURE (**C**) and T1-WI (**D**). **: *p* < 0.005, ***: *p* < 0.001.

**Figure 4:**
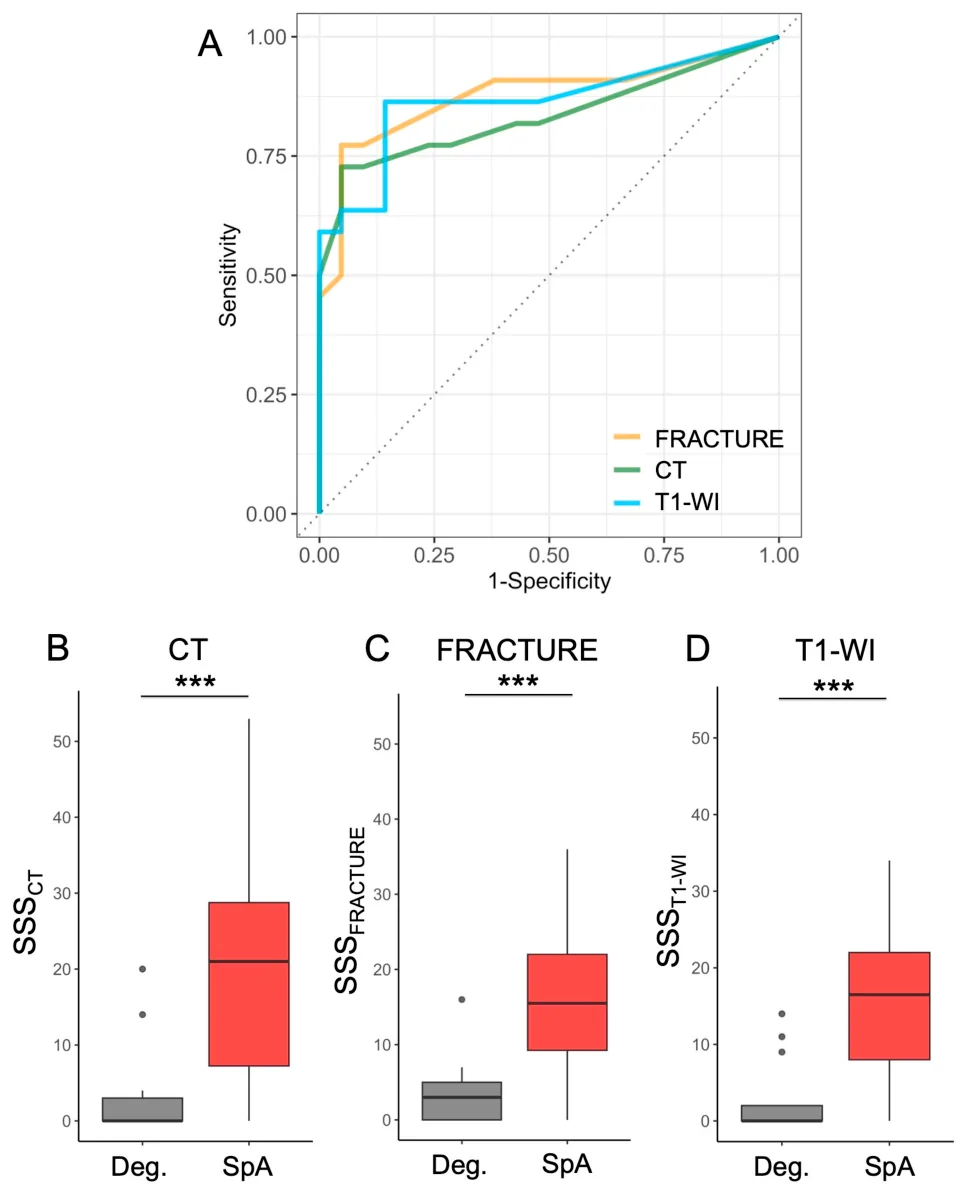
Performance of the consensus structural severity score (SSS) by Diekhoff et al. assessed on FRACTURE, CT and T1-weighted imaging (WI) to diagnose inflammatory sacroiliitis. (**A**) ROC curve for the 3 Likert scores. Boxplots showing the distribution of the 3 SSSs depending on the patient groups (i.e., spondyloarthropathy [SpA] and mechanical/degenerative [Deg.]) and the image type, i.e., CT (**B**), FRACTURE (**C**) and T1-WI (**D**). ***: *p* < 0.001.

**Figure 5:**
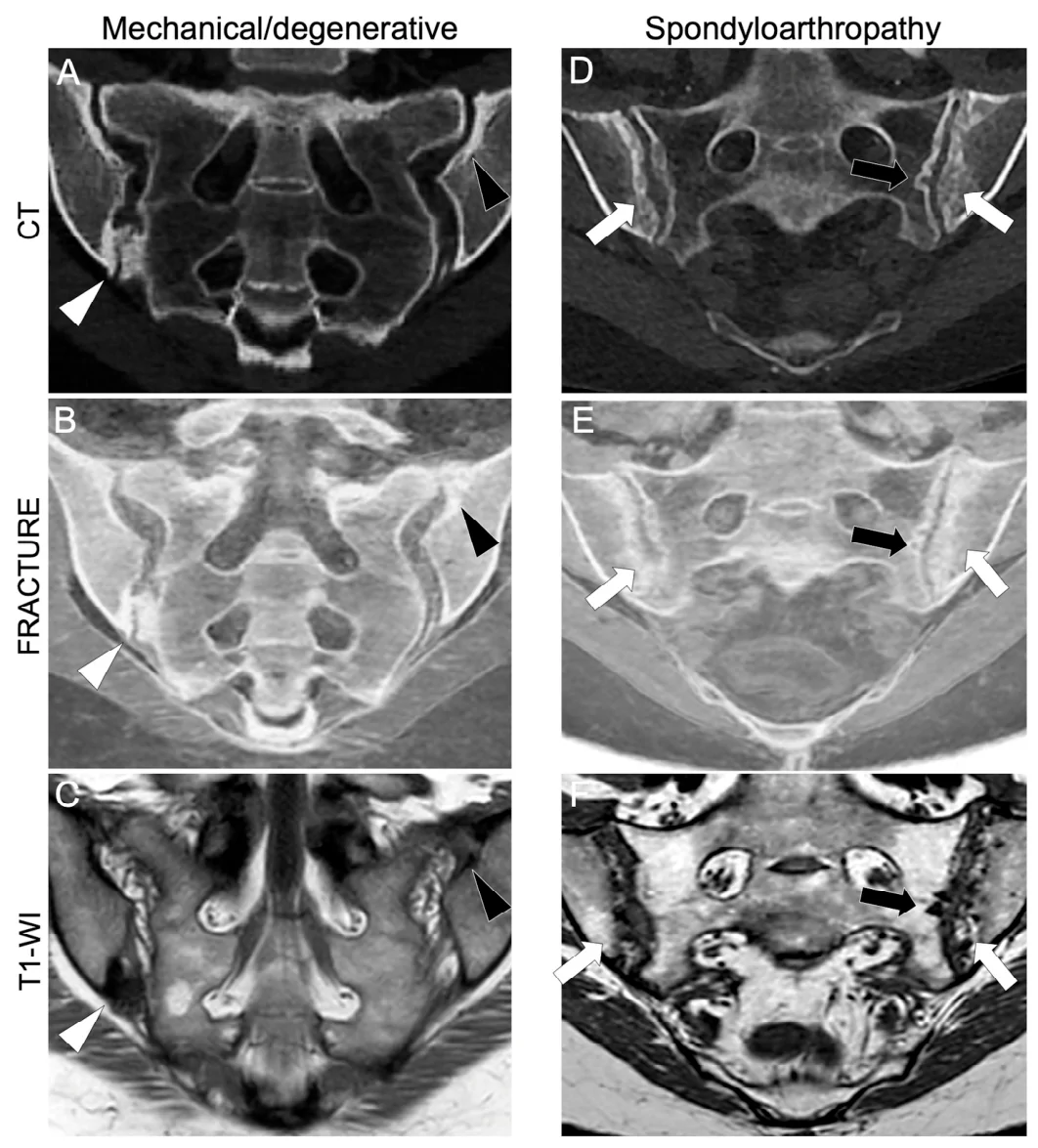
Correspondences between the 3 image types for the two possible diagnoses. The first patient corresponded to a 37-year-old, overweight woman who presented with chronic buttock pain suggestive of sacroiliitis that predominated on the right side. On coronal-oblique CT (**A**), a right degenerative and remodeled accessory articular facet of the posterior right sacroiliac joint was seen (white arrowhead), as well as a contralateral dense mechanical sclerosis (black arrowhead). While the correspondence was obvious on FRACTURE (**B**), it was hardly seen on T1-WI (**C**). All readers gave a Likert score of 0 regardless of the image type. The second patient corresponded to a 46-year-old man with a medical history of spondyloarthropathy, who presented with bilateral buttock pain. CT (**D**) showed extensive bilateral sclerosis (white arrows) with a right joint space enlargement and focal erosions notably on the sacral side of the left joint (black arrow). Again, the correspondence with FRACTURE (**E**) was good, but the sclerosis was less visible on T1-WI (**F**) and the erosions appeared enlarged and more confluent. All readers gave a Likert score of 2 regardless of the image type.

**Figure 6:**
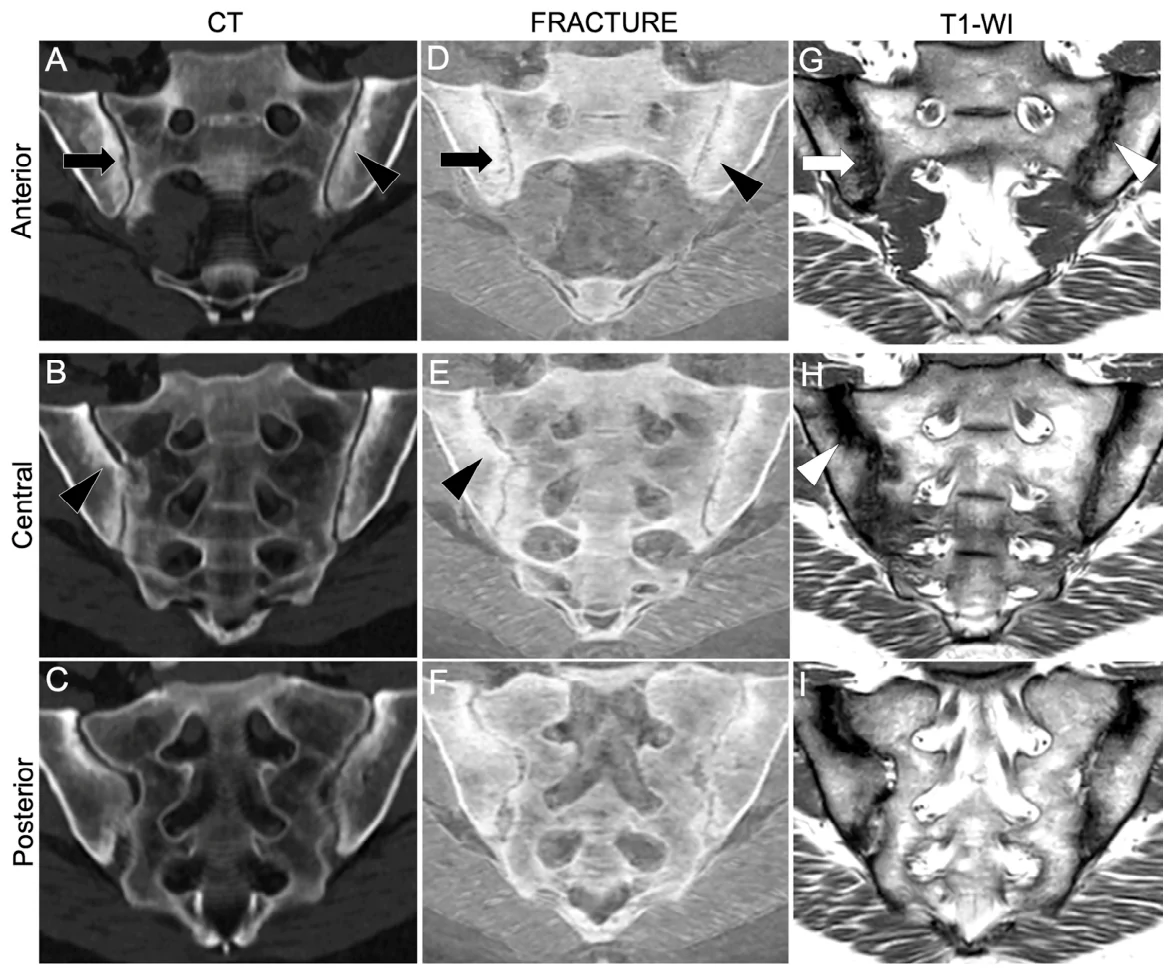
Visibility of the items of the structural severity score (SSS) on the 3 image types, i.e., CT (**A**–**C**), FRACTURE (**D**–**F**) and T1-weighted imaging (WI) (**G**–**I**), in the same 70-year-old woman with spondylarthropathy. The correspondence between sclerosis on CT and FRACTURE appeared strong on all sacroiliac locations (black arrowhead). However, the erosions were more difficult to delimit and confirm because of the irregularities due to noise of the joint space of the FRACTURE (black arrow). On T1-WI, the difficulties to distinguish sclerosis and large and confluent erosions, both with very low signal intensity on T1-WI, can be noticed (white arrow), and erosions on the left sacroiliac joint were over-estimated compared to CT (white arrowhead).

**Table 1:** Acquisition parameters of the MR sequences performed in the study.

Parameters	SPAIR T2-WI	DIXON T2-WI	T1-WI	FRACTURE
**Acquisition plane**	Coronal oblique	Coronal oblique	Coronal oblique	Coronal oblique
**Dimension**	2D	2D	2D	3D
**Readout type**	Cartesian, turbo spin echo	Cartesian, turbo spin echo	Cartesian, turbo spin echo	mFFE
**Field of View** (**mm**)	200 × 200	200 × 200	255 × 255	250 × 250
**Matrix**	640 × 640	384 × 384	576 × 576	640 × 640
**Acceleration** (**factor**)	SENSE (1.3)	compress sensing SENSE (1.4)	compress sensing SENSE (1.6)	compress sensing SENSE (7)
**In-plane spatial resolution** (**mm^2^**)	0.42 × 0.42	0.52 × 0.52	0.44 × 0.44	0.39 × 0.39
**Slice thickness** (**mm**)	3	3.5	3.5	0.55
**Flip angle**	90°	90°	90°	15°
**TE** (**ms**)	60	100 (ΔTE = 3.4 ms)	10	6 echos: 1st echo: 4.6 ms with ΔTE.6 ms between echos
**TR** (**ms**)	3167	2614	636	30
**No. of excitation**	1.6	1	1	1
**Acquisition time**	3 min 35 s	2 min 52 s	2 min 6 s	3 min 9 s
**No. of slices**	28	25	25	200

Note—Abbreviations: mFFE: multi-echo fast field echo, no.: number, SENSE: SENSitivity Encoding, SPAIR: Spectral Attenuated Inversion Recovery, TE: echo time, TR: repetition time, WI: weighted imaging.

**Table 2:** Study population.

Characteristics		Patients
**All patients**		
**Sex**		
	Men	20/43 (46.5)
	Women	23/43 (53.5)
**Age** (**years**)		
	Mean ± SD	46.5 ± 13.1
	Median [IQR] (range)	49 [37–52] (19–78)
**BMI** (**m^2^/kg**)		
	Mean ± SD	25.7 ± 5.5
	Median [IQR] (range)	26.3 [21.2–27.7] (16.7–41.5)
**Diagnosis**		
	Degenerative SIJ changes	21/43 (48.8)
	Inflammatory sacroiliitis	22/43 (51.2)
**SpA patients**		
**Etiology of inflammatory sacroiliitis**		
	Ankylosing spondylitis	20/22 (90.9)
	Psoariatic arthritis	2/22 (9.1)
**Time since diagnosis** (**years**)		
	Mean ± SD	8.3 ± 11.4
	Median [IQR] (range)	3 [1–10] (0.2–50)
**Peripheral symptoms** (**present**)		16/22 (72.7)
**Latest CRP** (**mg/L**)		
	Mean ± SD	10.3 ± 21
	Median [IQR] (range)	4 [0.5–6.6] (0–95)
**Treatments**		
	Ab anti-IL-17	1/22 (4.5)
	Ab anti-TNFalpha	13/22 (59.1)
	Methotrexate	3/22 (13.4)
	NSAID	5/22 (22.7)

Note—Data are numbers of patients with the percentages in parentheses for categorical data, or mean ± standard deviation (SD) and median, interquartile range (IQR) and range for numerical variables. Other abbreviations: Ab: antibody, CRP: C-reactive protein, IL: interleukin, NSAID: non steroid anti-inflammatory drug, SIJ: sacroiliac joint, SpA: spondyloarthropathy, TNF: tumor necrosis factor.

**Table 3:** Associations between the final diagnosis (inflammatory sacroiliitis vs. degenerative sacroiliac joint [SIJ] changes) and the non-radiological variables.

Characteristics		Inflammatory Sacroiliitis	Degenerative SIJ Changes	*p*-Value
**Sex**				0.8701
	Men	11/22 (50)	9/21 (42.9)	
	Women	11/22 (50)	12/21 (57.1)	
**Age** (**years**)				0.5758
	Mean ± SD	48.8 ± 14.4	44.1 ± 11.4	
	Median [IQR] (range)	49 [37.8–52] (19–57)	48 [37–52] (29–78)	
**BMI** (**m^2^/kg**)				0.0678
	Mean ± SD	24.2 ± 4.5	27.3 ± 6.1	
	Median [IQR] (range)	24.3 [20.8–26.6] (16.7–33.8)	26.6 [24.5–30.5] (17.7–41.5)	
**CRP** (**mg/L**)				0.0007 ***
	Mean ± SD	16.4 ± 25.4	0.9 ± 1.7	
	Median [IQR] (range)	6.1 [4–18] (1–95)	0 [0, 1] (0–5)	
**Duration of symptoms**				0.0042 *
	Mean ± SD	11.4 ± 11.4	5.1 ± 10.8	
	Median [IQR] (range)	10 [2–15.8] (0.2–40)	1.1 [0.3–4] (0.2–50)	
**Pain in other joints**				0.1442
	No	11/22 (50)	16/21 (76.2)	
	Yes	11/22 (50)	5/21 (23.8)	

Note—Data are numbers of patients with the percentages in parentheses for categorical data, or mean ± standard deviation (SD) and median, interquartile range (IQR) and range for numerical variables. Other abbreviations: CRP: C-reactive protein, SIJ: sacroiliac joint. Significant results are highlighted in bold. *: *p* < 0.05, ***: *p* < 0.001.

**Table 4:** Performances of the different possible binarizations of the Likert score to diagnose spondyloarthropathy-related sacroiliitis.

Modality/Sequence	Dichotomization	OR (95%CI)	*p*-Value	Accuracy (95%CI)	Sensitivity (95%CI)	Specificity (95%CI)
**CT**	Likert score = 1–2 (ref. 0)	10.5 (2.6–55.76)	0.0021 **	74.4 (58.8–86.5)	63.6 (40.7–82.8)	85.7 (63.7–97)
	Likert score = 2 (ref. 0–1)	Inf. (0–Inf.)	0.9943	**81.4** (**66.6–91.6**)	63.6 (40.7–82.8)	**100** (**83.9–100**)
**T1-WI**	Likert score = 1–2 (ref. 0)	25.33 (5.32–193.8)	0.0003 ***	**81.4** (**66.6–91.6**)	**72.7** (**49.8–89.3**)	90.5 (69.6–98.8)
	Likert score = 2 (ref. 0–1)	Inf. (0–Inf.)	0.9918	76.7 (61.4–88.2)	54.5 (32.2–75.6)	**100** (**83.9–100**)
**FRACTURE**	Likert score = 1–2 (ref. 0)	13.72 (3–100.72)	0.0023 **	74.4 (58.8–86.5)	59.1 (36.4–79.3)	90.5 (69.6–98.8)
	Likert score = 2 (ref. 0–1)	Inf. (0–Inf.)	0.9930	69.8 (53.9–82.8)	40.9 (20.7–63.6)	**100** (**83.9–100**)

Note—Abbreviations: 95%CI: 95% confidence interval, OR: odds ratio, ref.: level of reference, WI: weighted imaging. For OR, sensitivity, specificity and accuracy, the highest value is highlighted in bold. **: *p* < 0.005, ***: *p* < 0.001. Significant *p*-values are also in bold.

**Table 5:** Correlations between structural severity scores (SSS, and its items, namely erosions, sclerosis and joint space alterations) assessed on CT (i.e., modality of reference) and T1-weighted imaging (WI) or FRACTURE sequence.

Reference	Correlation with	Spearman Rho (95%CI)	*p*-Value
**SSS_CT_**	SSS_T1-WI_	0.774 (0.668–0.988)	<0.0001 ***
	SSS_FRACTURE_	**0.814** (**0.737–0.954**)	<0.0001 ***
**SSS_CT,erosions_**	SSS_T1-WI,erosions_	**0.714** (**0.574–0.936**)	<0.0001 ***
	SSS_FRACTURE,erosions_	0.526 (0.288–0.757)	0.0002 ***
**SSS_CT,sclerosis_**	SSS_T1-WI,sclerosis_	0.503 (0.323–0.71)	0.0005 ***
	SSS_FRACTURE,sclerosis_	**0.679** (**0.474–0.902**)	<0.0001 ***
**SSS_CT,joint space_**	SSS_T1-WI,joint space_	0.562 (0.364–0.848)	<0.0001 ***
	SSS_FRACTURE,joint space_	**0.715** (**0.555–0.984**)	<0.0001 ***

Note—Other abbreviations: 95%CI: 95% confidence interval. Highest rho value is emphasized in bold. ***: *p* < 0.001. Significant results are in bold.

**Table 6:** Inter- and intra-observer reproducibility analysis.

Characteristics	Reproducibility Type	Sequence	Value ^§^ (95%CI)	*p*-Value
SSS	Inter-observer	T1-WI	**0.759** (**0.578–0.869**)	<0.0001 ***
		FRACTURE	0.741 (0.551–0.859)	<0.0001 ***
	Intra-observer (Reader-1)	T1-WI	**0.826** (**0.688–0.907**)	<0.0001 ***
		FRACTURE	0.727 (0.529–0.850)	<0.0001 ***
	Intra-observer (Reader-2)	T1-WI	0.903 (0.820–0.949)	<0.0001 ***
		FRACTURE	**0.935** (**0.877–0.966**)	<0.0001 ***
Likert score	Inter-observer	T1-WI	**0.799** (**0.633–0.975**)	<0.0001 ***
		FRACTURE	0.762 (0.613–0.96)	<0.0001 ***
	Intra-observer (Reader-1)	T1-WI	**0.759** (**0.587–0.941**)	<0.0001 ***
		FRACTURE	0.632 (0.44–0.866)	<0.0001 ***
	Intra-observer (Reader-2)	T1-WI	0.865 (0.731–1)	<0.0001 ***
		FRACTURE	**0.899** (**0.798–1**)	<0.0001 ***

Note. ^§^ Value correspond to intra-class correlation coefficient for structural severity score (SSS) and weighted Kappa for Likert score. Highest reproducibility value is emphasized in bold. Other abbreviations: 95%CI: 95% confidence interval, WI: weighted imaging. ***: *p* < 0.001. Significant results are in bold.

**Table 7:** Summary of the main advantages and limitations of the FRACTURE sequence identified in the present study.

Advantages of FRACTURE	Disadvantages of FRACTURE
Non-ionizing technique, avoiding radiation exposure associated with CT: relevant in young SpA patients and in the context of repeated imaging	Lower performance for erosion detection compared with T1-WI in this study, with difficulty distinguishing erosions from degenerative subchondral cysts
Provides CT-like structural bone contrast within the MRI examination, allowing structural assessment without an additional CT examination (reduced cost)	Lower agreement with CT for erosions than T1-WI (although not statistically significant)
High spatial resolution enabling detailed assessment of SIJ structural abnormalities	Potentially increased image noise related to the highly accelerated 3D GRE acquisition
3D isotropic acquisition allows multiplanar reconstructions in any orientation, which may be useful with atypical SIJ morphology or suboptimal standard imaging planes	Multiplanar reconstructions, particularly oblique coronal reformations, may appear less sharp than directly acquired T1-WI
Showed good correlation with CT for overall structural severity score, which was numerically higher than T1-WI (although not statistically significant)	Slightly lower reproducibility than T1-WI in this study
Demonstrated good performance for structural severity assessment	Limited reader familiarity with FRACTURE may have influenced lesion detection and reproducibility
Detected additional lumbosacral neojoints and accessory articular facets not identified on T1-WI	Susceptibility artifacts may impair interpretation, particularly near air-containing structures and metallic implants
GRE-based implementation may be more readily integrated into conventional clinical MRI workflows than UTE/ZTE approaches requiring dedicated sequence implementations	Vendor-specific implementation in the present study may limit generalizability and availability across MRI platforms

Note—abbreviations: CT: computed tomography, GRE: gradient echo, SIJ: sacroiliac joint, UTE: ultra-short echo time imaging, WI: weighted imaging, ZTE: zero echo time imaging.

**Figure A1:**
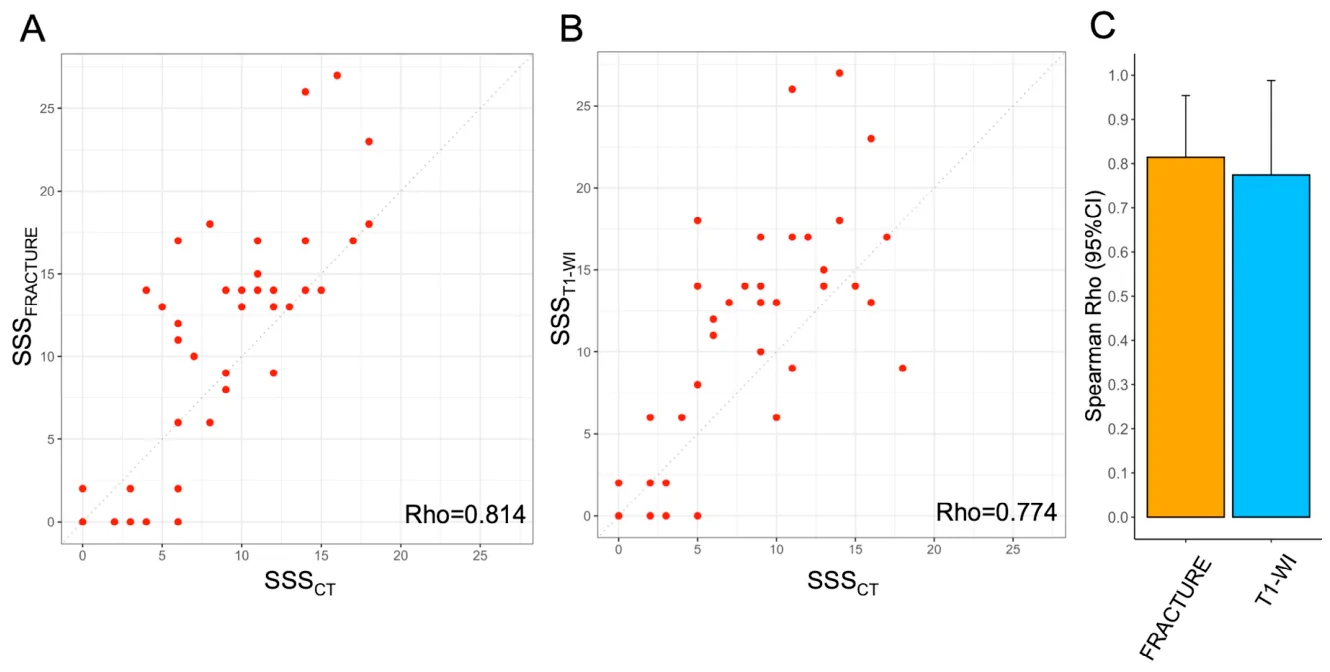
Correlations between the structural severity score (SSS) assessed with the reference image type (CT) and assessed with FRACTURE (**A**) and T1-weighted imaging (WI) (**B**). Each red point corresponds to a sacroiliac joint from one of the 22 patients with spondylarthropathy (i.e., 44 points). Rho corresponds to the correlation coefficient evaluated with the Spearman rank test. (**C**) Comparison between the Rho values for FRACTURE and T1-WI with 95% confidence interval (95%CI) showing no statistical difference (*p* = 0.7850).

**Figure A2:**
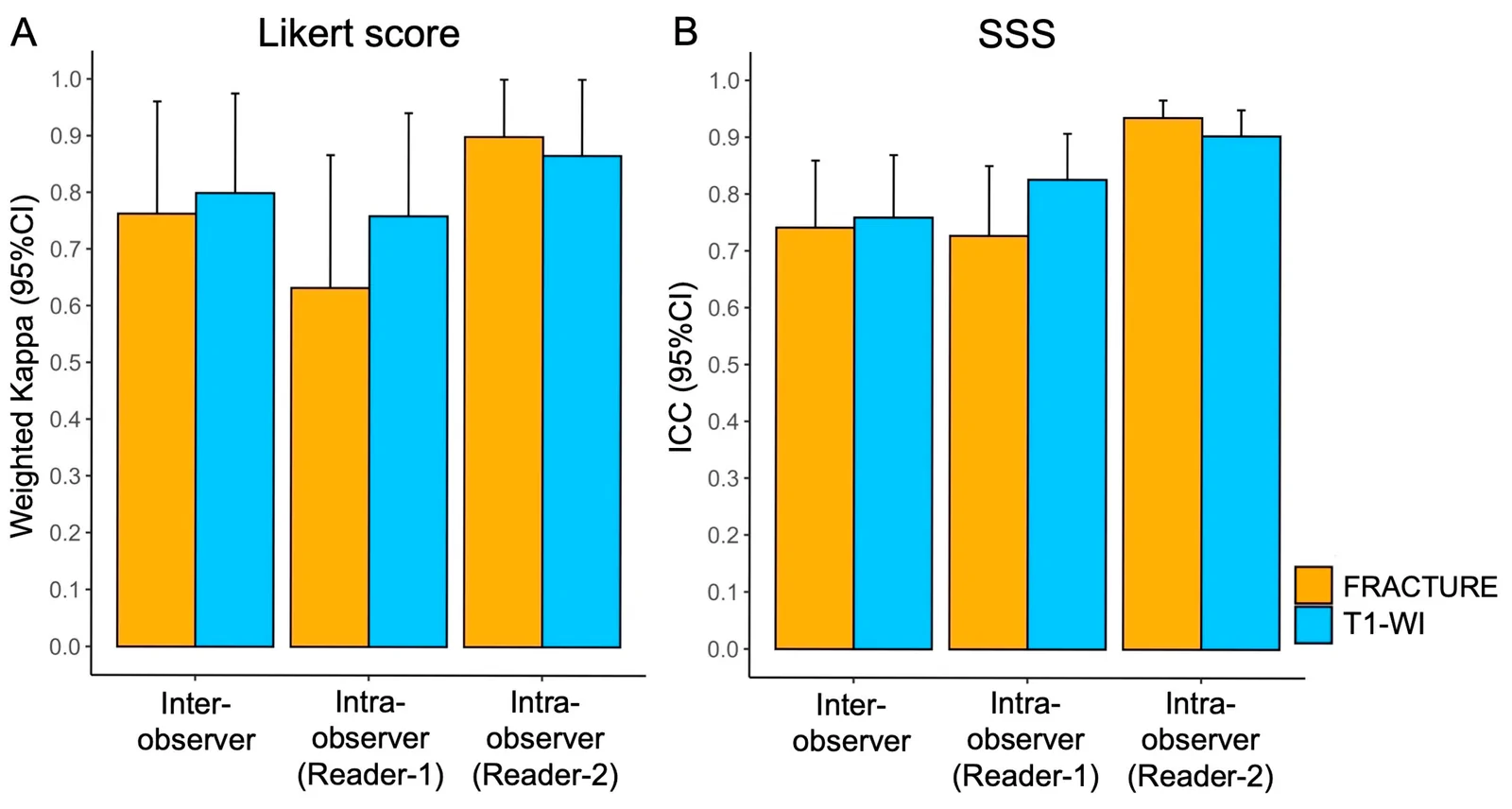
Summary of the reproducibility analysis for (**A**) the Likert scores (ordinal variables, i.e., estimated with weighted Kappa) and (**B**) structural severity scores (SSS, i.e., estimated with intra-class correlation coefficient [ICC]). Results are given with 95% confidence interval (95%CI).

## Data Availability

The raw data and R scripts supporting this study are available from the corresponding author on reasonable request.

## Abbreviations

The following abbreviations are used in this manuscript:

AUROC	area under the ROC curve
BMI	body mass index
CI	confidence interval
GRE	gradient echo
ICC	intra-class correlation coefficient
IQR	interquartile range
Kw	weighted Kappa value
OR	odds ratio
SD	standard deviation
SI	signal intensity
SIJ	sacroiliac joints
SSS	structural severity score
WI	weighted imaging

## Appendix A

**Table A1:** Associations between the final diagnoses and the Likert scores assessed independently on the 3 image types, i.e., CT, FRACTURE and T1-weighted imaging (WI).

Characteristics	Degenerative Sacroiliitis	Inflammatory Sacroiliitis	*p*-Value
**CT Likert score**			**<0.0001 *****
0	18/21 (85.7)	8/22 (36.4)	
1	3/21 (14.3)	0/22 (0)	
2	0/21 (0)	14/22 (63.6)	
**FRACTURE Likert score**			**0.0013 ****
0	19/21 (90.5)	9/22 (40.9)	
1	2/21 (9.5)	4/22 (18.2)	
2	0/21 (0)	9/22 (40.9)	
**T1-WI Likert score**			**<0.0001 *****
0	19/21 (90.5)	6/22 (27.3)	
1	2/21 (9.5)	4/22 (18.2)	
2	0/21 (0)	12/22 (54.5)	

Note—Data are numbers of patients with the percentages in parentheses. Tests are Chi-square tests. **: *p* < 0.005, ***: *p* < 0.001. Significant results are in bold.

## Appendix B

**Table A2:** *p*-values of the pairwise comparisons of diagnostic accuracy to diagnose the type of sacroiliitis. The upper table corresponded to Likert scores binarized as 2 vs. 0–1 (reference level). The lower table corresponded to Likert scores binarized as 1–2 vs. 0 (reference level).

	CT Likert Score = 2	FRACTURE Likert Score = 2	T1-WI Likert Score = 2
**CT Likert score = 2**	-	*p* = 1306	*p* = 0.6171
**FRACTURE Likert score = 2**	-	-	*p* = 0.3711
**T1-WI Likert score = 2**	-	-	-
	**CT Likert Score = 1 or 2**	**FRACTURE Likert Score = 1 or 2**	**T1-WI Likert Score = 1 or 2**
**CT Likert score = 1 or 2**	-	*p* = 1.000	*p* = 0.3710
**FRACTURE Likert score = 1 or 2**	-	-	*p* = 0.4497
**T1-WI Likert score = 1 or 2**	-	-	-
